# Intermediate care at a community hospital as an alternative to prolonged general hospital care for elderly patients: a randomised controlled trial

**DOI:** 10.1186/1471-2458-7-68

**Published:** 2007-05-02

**Authors:** Helge Garåsen, Rolf Windspoll, Roar Johnsen

**Affiliations:** 1 Department of Public Health and General Practice, Faculty of Medicine, The Norwegian University of Science and Technology (NTNU), 7491 Trondheim, Norway; 2 St. Olavs University Hospital, 7006 Trondheim, Norway

## Abstract

**Background:**

Demographic changes together with an increasing demand among older people for hospital beds and other health services make allocation of resources to the most efficient care level a vital issue. The aim of this trial was to study the efficacy of intermediate care at a community hospital compared to standard prolonged care at a general hospital.

**Methods:**

In a randomised controlled trial 142 patients aged 60 or more admitted to a general hospital due to acute illness or exacerbation of a chronic disease 72 (intervention group) were randomised to intermediate care at a community hospital and 70 (general hospital group) to further general hospital care.

**Results:**

In the intervention group 14 patients (19.4%) were readmitted for the same disease compared to 25 patients (35.7%) in the general hospital group (p = 0.03). After 26 weeks 18 (25.0%) patients in the intervention group were independent of community care compared to seven (10.0%) in the general hospital group (p = 0.02). There were an insignificant reduction in the number of deaths and an insignificant increase in the number of days with inward care in the intervention group. The number of patients admitted to long-term nursing homes from the intervention group was insignificantly higher than from the general hospital group.

**Conclusion:**

Intermediate care at a community hospital significantly decreased the number of readmissions for the same disease to general hospital, and a significantly higher number of patients were independent of community care after 26 weeks of follow-up, without any increase in mortality and number of days in institutions.

## Background

An increasing demand among elderly for hospital beds and other health services make allocation of resources to the most efficient care level a vital issue [1]. In 1995 there were 42.8 admissions to general and university hospitals per 100 persons above 80 years in Norway. This increased by more than 40% to 60.8 in 2005 [2].

Both in UK and Norway there is a particular challenge of Payment by Results where tariffs in general and university hospitals are set on a diagnosis and procedure-based system, which does not account for increased lengths of stay for patients with physical disability [3,4]. In the UK the number of persons with physical disability and a high level of need of care are estimated to increase by 54% by 2025, most of these will be older persons [5]. In addition to the loss of health and function for the patients and the social and economic burden for their families, this increased need of care is considered to be a major economic challenge for societies worldwide [1].

Moreover, fragile elderly patients often have to stay at general hospitals after the treatment of the acute illness is completed due to lack of a stable social network, lack of familial or municipal capacity to deliver proper care in their own homes or shortage of suitable nursing home beds [6].

The health care provision in Norway is based on a decentralised model [7]. The municipalities (primary health care) are responsible for home care services, nursing homes, community hospitals, family physicians, health cervices for mothers, children and youth, midwives, physiotherapists, occupational therapists and emergency services. The government (secondary health care) owns and runs district general hospitals, university hospitals and ambulance services through regional health authorities (five regions).

In 2001 an intermediate care department was established at a teaching nursing home (community hospital) [8] located in the city of Trondheim, Norway to perform intermediate care [9] for older patients initially admitted at the city general hospital, but without any need for further advanced hospital care. The goal was to create a department functioning as a new link between advanced care at a general hospital and community home care to optimise recovery before returning home after general hospital care [7]. There is little published knowledge about patient outcome and cost effectiveness when intermediate care [9] is provided at a community hospital instead of standard care at a general hospital [10-14].

### Aims

The aim was to test the hypothesis that intermediate care at a community hospital compared to traditional prolonged care at a general hospital would reduce morbidity assessed as number of readmissions for the same disease to the general hospital, need of home care services and long-term nursing homes without increasing mortality and the number of days in institutions.

## Methods

### Setting

Twenty beds at Søbstad Nursing Home were re-assigned in late 2002 to be a community hospital performing intermediate care, which included increased numbers of trained nurses from 12.5 to 16.7 man-labours per week and doctors' hours, performed by three general practitioners, from 7 hours to 37.5 hours per week. All employees underwent a training programme provided by the general hospital. The department was also upgraded with laboratory facilities including intravenous pumps, equipment for continuously monitoring of oxygen-saturation in blood, laboratory equipment to measure infectious variables, hemoglobin and glucose in blood. Other blood tests could be delivered each day to the main laboratory at the general hospital with answers provided within the same working day.

The city general hospital in Trondheim, St. Olavs University Hospital, is both a general hospital for the municipality of Trondheim and a university hospital for the three counties in Mid-Norway. In this trial the function as a general hospital was included.

### Intermediate care intervention

The experimental intervention was based on individualised intermediate care including evaluation and treatment ("care" and "cure") of each patient's diseases [13]. However, the main focus was to improve the patients' ability to manage daily activities when returning home.

On admission to the community hospital the physicians performed a medical examination of the patients and a careful evaluation of available earlier health records from the admitting general practitioner, the general hospital physicians and the community home care services. The communication with each patient and his family focusing on physical and mental challenges was also essential to understand the needs and level of care.

The care at the different departments at the general hospital and the communication with primary health care followed the standard routines through the formal organisation.

### Trial design

Intermediate care at the community hospital was compared to conventional care in general hospital beds at medical, surgical and orthopedic departments.

Before the trial started participating physicians and nurses at the general hospital together with general practitioners and community nurses developed inclusion criteria through a Delphi technique [15]. One of the authors (HG) facilitated requests for proposals and organised the proposals received, and was responsible for communication between the participants. Eventually, there were four inclusions criteria as eligible participants should be; 1) patients aged 60 years or more admitted the general hospital due to an acute illness or an acute exacerbation of a known chronic disease, 2) probably be in need of inward care for more than three to four days, 3) admitted from their own homes and 4) expected to return home when inward care was finished. Exclusion criteria were severe dementia or a psychiatric disorders needing specialised care 24 hours a day.

When an eligible patient was identified and accepted for inclusion, a blinded randomisation was performed by the Clinical Research Department at the Faculty of Medicine using random number tables in blocks to ensure balanced groups.

The number of deaths was monitored continuously during the whole trail as it was decided prior to the study that an increase in number of deaths at the community hospital should terminate the study.

Outcome variables were number of readmissions for the same disease, need of community home care and need of long-term nursing home. Readmissions for the same disease, according to the national definition, are defined as acute, non-planned admissions within 60 days for the same disease. Number of days in institutions after randomisation, number of deceased patients and days before death were assessed as well. All data were collected by one of the authors, (HG), according to prepared schemes from patients' medical records at the hospitals and at primary health services. The assessments of days in institution, readmissions and cause-specific deaths were monitored through the patient administrative systems, independent of treatment groups.

Two specially trained nurses monitored physical functioning (ADL) on 72 items with scores from one to four in each item, both at the intermediate department and at the general hospital, by a national system, Gerix [16]. With an average ADL of one the patient is functioning perfectly in all areas, whereas an average score of four indicates a need of excessive help and care in all aspects of daily living. ADL was assessed for all patients prior to the inclusion to the trial, and the ADL was used as covariate or confounder in the multivariate analysis. General hospital doctors set the diagnosis at all patients prior to randomisation.

### Approval

The Regional Committee for Medical Research Ethics for Central Norway approved the study, the patient information and the consent schemes. The study was granted license by the Norwegian Data Inspectorate to process personal health data. Each participating patient signed a written informed consent formula at the general hospital prior to the inclusion to the study.

### Statistical analysis

The sample size was estimated to detect a difference of 25 per cent in the number of readmissions for the same disease, as an assessment of morbidity, between the groups with alpha 0.05 and power of 0.80. To achieve this we needed 65 patients in each group, altogether 130 patients.

All data are presented an analysed according to the CONSORT checklist (see Additional file 1). The comparisons between the intervention and control group were analysed as intention-to-treat analyses according to the CONSORT instructions. Some results from treatment analyses, dependent on where the patient received his treatment, are also presented.

We undertook all analyses using SPSS version 14.0. for Windows. Survival curves were estimated by Kaplan-Meier. The distribution of continuous variables was tested by comparing means and medians and by normality plots. Differences in number of patients with readmissions for the same disease and need of home care services or nursing homes between groups were tested by chi square tests, and differences in mean number of days in institution were tested both by paired t-test and by Wilcoxon signed rank test. Differences in readmissions and need of home care or nursing home were also analysed in logistic models adjusted for gender, age, ADL score and diagnosis. Hosmer and Lemeshows goodness of fit test tested the fit of the logistic models. The number of days in institution was compared between groups using covariance analyses with age, gender, ADL scores and diagnoses as covariates. The level of significance was set to p = 0.05.

## Results

**Figure 1:**
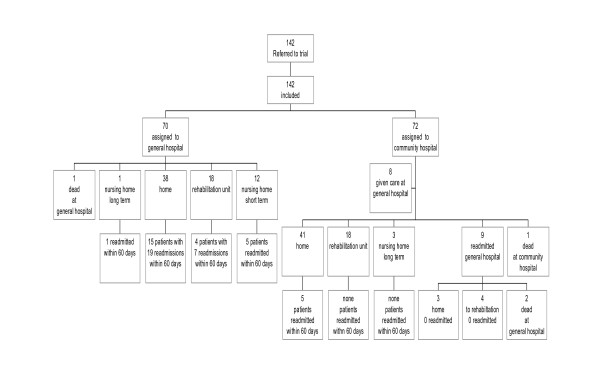
Trial profile and flow chart the first 60 days.

From August 2003 until the end of May 2004 142 patients were eligible for inclusion and 70 were randomised to continued care at the general hospital (general hospital group) and 72 to the community hospital (intervention group) (Figure 1). All patients randomised for care at the community hospital were transferred from the general hospital within 24 hours after the time of inclusion to the study and immediately after the time of randomisation. Sixty-four patients were transferred from the general hospital to intermediate care (intermediate care group), as eight of the patients randomised for intervention were never transferred due to an acute and severe deterioration of their medical conditions after inclusion. In the intention-to-treat analyses they were included in the intervention group, otherwise, in the treatment-analyses they were dealt with as a separate group. There were no dropouts, except for deaths, during the trial and for all patients all data were collected from the first day at the general hospital and until the end of the trial or at the time of death.

### Patient characteristics

At randomisation (index day), the patients randomised to intermediate care or to general hospital care were comparable with respect to number of days of care before randomisation, mean and median age, diagnosis, gender, ADL and matrimonial status (Table 1).

**Table 1:** Baseline characteristics. Trondheim 2003–4.

	**Assigned community hospital**		**Assigned general hospital**
	
	Intermediate care group (n = 64)	Intervention group (n = 72)	General hospital group (n = 70)
***Demography***			
**Gender**			
Males	14 (21.9%)	20 (27.8%)	27 (38.6%)
Females	50 (78.1%)	52 (72.2%)	43 (61.4%)
**Age males**			
Mean (SD)	79.5 (1.5)	80.6 (1.1)	78.4 (1.2)
Median	79.0	80.0	79.0
**Age females**			
Mean (SD)	81.4 (1.1)	80.6 (1.1)	83.1 (1.0)
Median	82.5	82.0	83.0
**Age both genders**			
Mean (SD)	80.9 (0.9)	80.6 (0.8)	81.3 (0.8)
Median	81.5	81.5	81.0
**Living with spouse**			
Males	7	10	9
Females	6	6	6
			
***ADL-scores***			
**Both genders**			
Mean (SD	2.19 (0.1)	2.24 (0.9)	2.05 (0.7)
Median	2.13	2.29	2.02
**Males**			
Mean (SD	2.30 (0.2)	2.42 (0.9)	2.08 (0.1)
Median	2.37	2.37	2.00
**Females**			
Mean (SD)	2.17 (0.1)	2.24 (0.8)	2.05 (0.1)
Median	2.10	2.18	2.03
			
***Primary diagnoses***			
Cardiological diseases	21 (32.8%)	22 (30.6%)	20 (28.6%)
Infections	7 (10.9%)	13 (18.1%)	16 (22.9%)
Fractures/contusions	13 (20.3%)	14 (19.4%)	12 (17.1%)
Pulmonary diseases	5 (7.8%)	5 (6.9%)	6 (8.6%)
Neurological diseases	5 (7.8%)	5 (6.9%)	4 (5.7%)
Cancers	2 (3.1%)	2 (2.8%)	4 (5.7%)
Psychiatric diseases	1 (1.6%)	1 (1.4%)	0 (0%)
Other diseases	10 (15.6%)	10 (13.9%)	8 (11.4%)

The general hospital group had the best mean ADL, 2.05, and the intervention group somewhat worse with a mean score at 2.24, a non-significant difference (p = 0.27). The eight patients not transferred to intermediate care, due to their medical condition, had a more severe loss in ADL, mean score 2.60.

### Readmissions for the same disease

Fourteen patients (19.4%) in the intervention group were readmitted for the same disease. Nine (64.3%) of these readmissions took place while the patients were at the department and five (35.7%) after discharge to their homes. Of the patients in the general hospital group 25 patients (35.7%) were readmitted, comprising 32 readmissions. Nineteen (76.0%) of these patients were readmitted after discharge to their homes and six (24.0%) during care at rehabilitation departments. OR for readmissions for the same disease in the intervention group versus the general hospital group was 2.77 (95% CI 1.18–6.49) (Table 3). There was statistically a significant difference between the two groups (p = 0.03). In a multivariate analysis, adjusted for gender, age, diagnosis and ADL score, there was also a significant difference (p = 0.02). In a treatment-analysis there was still a significant difference (p = 0.02).

### Need of nursing homes and home care after six months

Six months after discharge from intermediate care or from care at the general hospital 38 patients (52.8%) in the intervention group and 44 patients (62.9%) in the general hospital group needed home care, a non-significant difference. The OR for the need of home care was 1.21 (95% CI 0.59–2.52) in the intervention group versus the general hospital group (Table 3).

Eighteen (25.0%) patients in the intervention group were independent of home care compared to seven (10.0%) in the general hospital group (p = 0.02) (Table 2). The OR was 0.31 (95% CI 0.11–0.88) in favour of the intervention group. In the treatment-analysis the differences was still statistically significant (p = 0.02).

**Table 2:** Numbers of readmissions for the same disease, deaths, need of nursing homes and home care. *P-values *based on comparisons between intervention and general hospital groups according to intention-to-treat analyses. Trondheim 2003–4.

	**Assigned community hospital**		**Assigned general hospital**		
	
	Intermediate care group (n = 64)	Intervention group (n = 72)	General hospital group (n = 70)	p-values	*adjusted p^1^*
Readmissions^2^	13 (20.3%)	14 (19.4%)	25 (35.7%)	0.03	*0.02*
Deaths	8 (12.5%)	9 (12.5%)	14 (20%)	0.23	*0.15*
Nursing homes^3^	7 (10.9%)	7 (9.7%)	5 (7.1%)	0.45	*0.76*
Home care	32 (50.0%)	38 (52.8%)	44 (62.9%)	0.22	*0.37*
No care	17 (26.6%)	18 (25.0%)	7 (10.0%)	0.02	*0.01*

^1 ^Adjusted for age, gender, ADL, diagnosis

^2 ^Readmissions for the same disease

^3 ^Long-term nursing homes

Twelve patients, seven (9.7%) from the intervention and five (7.1%) from the general hospital group, were living at long-term nursing homes, a non-significant difference, and the OR between the intervention and hospital groups were 2.19 (95% CI 0.51–9.40).

### Number of days of care after randomisation

Patients in the intervention group stayed on average 17.5 days (95% CI 14.6–20.4) for initial intermediate care, 10.4 days (95% CI 5.6–15.2) at rehabilitation departments and 3.1 days (95% CI 1.2–5.0) at the general hospital due to readmissions for the same disease, giving a total average of number of days with inward care after the index day of 31.0 days (95% CI 26.1–34.7) (Table 4). Patients in the general hospital group stayed 9.1 days (95% CI 6.9–11.2) at the general hospital for initial care, 13.1 days (95% CI 8.2–18.1) at various rehabilitation departments and were readmitted 7.6 days (95% CI 3.6–11.6) at the general hospital, giving a total of the number of 29.8 days (95% CI 23.2–36.4) with inward care after the index day.

**Table 3:** The risks of readmissions for the same disease, deaths, need of nursing home, and the use of home care assessed as OR between intervention (0) and general hospital group (1) according to intention-to-treat analyses with 95% Confidence Intervals. Trondheim 2003–4.

	**OR**	**95% CI**
Readmissions for the same disease	2.77	1.18–6.49
Deaths	1.91	0.72–5.01
Long-term nursing homes	2.19	0.51–9.40
Home care	1.21	0.59–2.52
No public care	0.31	0.11–0.88

**Table 4:** Number of days (with 95% Confidence Intervals) in institution after randomisation. Trondheim 2003–4.

	**Assigned community hospital**		**Assigned general hospital**		
	
	Intermediate care group (n = 64)	Intervention group (n = 72)	General hospital group (n = 70)	p-values	*adjusted p^1^*
Number of days before randomisation^2^	10.6 (9.0–12.1)	10.7 (9.2–12.1)	10.0 (8.2–11.8)	0.6	*0.8*
Number of days initial care	17.9 (14.7–21.1)	17.5 (14.6–20.4)	9.1 (6.9–11.2)	0.00	*0.00*
Days at rehabilitation units	9.6 (4.9–14.2)	10.4 (5.6–15.2)	13.1 (8.2–18.1)	0.43	*0.22*
Number of readmission days^2^	3.3 (1.2–5.4)	3.1 (1.2–5.0)	7.6 (3.6–11.6)	0.04	*0.02*
Total number of days of inward care	30.8 (25.2–36.3)	31.0 (26.1–34.7)	29.8 (23.2–36.4)	0.79	*0.80*

^1^Adjusted for age, gender, ADL score and diagnosis

^2 ^Readmissions at general hospital for the same disease

There was a non-significant difference in the total number of days with inward care between the patients' groups (p = 0.79), (paired t-test, using Wilcoxon signed rank test did not change the level of significance). Adjusting number of days of care for gender, age, ADL and diagnosis, there was still an insignificant difference in number of days at the institutions between the groups (p = 0.80). However, there was a significant difference in number of days of initial care in favour of the general hospital group (p = 0.00), and in number of readmission days in favour of the intervention group (p = 0.04) (Table 4).

### Mortality within six months

**Figure 2:**
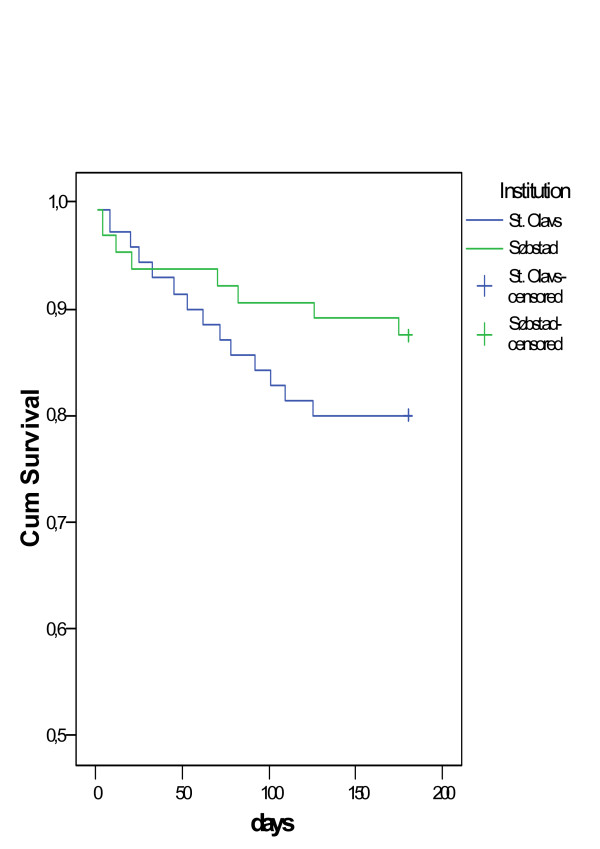
**Accumulated survival rates**. Survival time after intermediate care (Søbstad): 165 days (95% CI 154–176) Survival time after general hospital care (St. Olavs): 156 days (95% CI 144–165)

Twenty-three patients, nine (12.5%) in the intervention group and 14 (20%) in the general hospital group, died within six months (Table 2, Figure 2), a non-significant difference (p = 0.23). There were no differences between males (17.0% deceased) and females (16.1% deceased). In a treatment-analysis the difference in number of deaths was still statistically insignificant.

## Discussion

This trial demonstrated that elderly patients with acute diseases or deterioration of a chronic disease initially handled at a general hospital and subsequently offered intermediate care, had lower readmission rates (p = 0.03), and had a higher number of patients independent of community care (p = 0.02) than patients given traditional prolonged care at a general hospital. The differences in total number of days with inward care were minor. The differences in number of deaths and need of home care were in favour of the intervention group, however, statistically insignificant.

All patients were transferred immediately after randomisation to the community hospital except the eight patients with a severe and acute deterioration of their disease. These patients could have been treated as readmissions for the same diseases in the intention-to-treat analyses. However, the decisions not to transfer these patients were undertaken by the physicians at the general hospital and not by the physicians at the community hospital. Treated as readmissions in the statistical analyses resulted in an insignificant reduction of the number of readmissions (p = 0.14, adjusted p = 0.11) and an insignificant difference in number of days readmitted in favour of the intervention group; 4.4 (95% CI 2.6–6.9) days versus 7.6 (95% CI 3.6–11.6) days.

The present study appears to be the first randomised controlled trial where included patients have been an unselected general hospital population above 60 years of age. Another strength of this trial was that all patients received the same optimal care in the initial stage of their illness before randomisation.

As one of the authors, blinded for which group the patients belonged to, collected all information from medical records and from the patient administrative systems, information bias by collection was possible. As all data was objective measures as readmissions for the same disease, use of home care and number of deaths, the registration was considered to be accurate.

Several efforts have been developed to reduce number of days of inward care and to facilitate discharge from general hospitals including discharge planning, nurse led inpatient care, hospital at home regimes, general practitioners hospitals, community hospitals and patients hotels [10]. Some studies have found a better functional outcome and reduced mortality when older patients were treated at specialised geriatric wards [17-19], whilst the benefit of early supported discharge of stroke patients was ascribed a structured collaboration between primary and secondary health care [20,21].

Several community hospitals in Norway are comparable with community hospitals in England [7,8] and general practitioners hospitals in Holland [23] where some studies have explored their appropriateness [11,12,22-25]. In Norway the use of nursing homes and community hospitals may have been overlooked as appropriate alternatives, and research on such models has been sparse both nationally and internationally [7,22].

A limitation of performing intermediate care is the lack of possibility to identify which of the components that are working so well. However, some of the main components in the intervention were assessments of ADL and a consecutive and closely communicating and cooperating with each patient and his social and professional networks to identify the best supportive solutions. This communication, including the continuous dialogue with the rest of the primary health care in the municipality, was probably the central element of the care that seems to be efficient in reducing the number of readmissions for the same disease, the need of community care and allowing the professional teams to optimise the follow-up after discharge.

The communication process is always complex. Older people are a more heterogeneous group than younger people, and maybe they have experienced several more or less successful diagnosing and treatment procedures. Health personal and older people can have different perception of what are illness and the consequences of illness. As a consequence, unclear communication can cause the whole medical encounter to fall apart.

Intermediate care at a community hospital seems to be highly effective.

In a modern health care system care is more and more specialised, fragmented and organ-focused. In addition to the expansion of further sub-specialising in modern medicine, the results from this study underscore the additional need of better step-down care systems at an intermediate level. It is indeed relevant to question the appropriateness of prolonged traditional general hospital care for this rapidly increasing group of patients.

There are little existing scientific evidence of the benefits of intermediate care [26] and more randomised controlled trials are necessary to test different models for intermediate care at community hospitals as alternatives to general hospital admissions and as alternatives to prolonged general hospital care to confirm any benefits of intermediate care. Additionally, the economic consequences have to be explored.

## Conclusion

Intermediate care at a community hospital compared to ordinary prolonged care at a general hospital, reduced significantly the number of readmissions for the same disease to the general hospital and increased significantly the number of patients being independent of community care after 26 weeks of follow-up, with an insignificant increase in the number of days in institutions and without any increase in mortality. Regarding morbidity and mortality after 26 weeks of follow-up, the results favors alternative intermediate care at primary level.

## Competing interests

The author(s) declare that they have no competing interests.

## Authors' contributions

HG and RJ developed the idea of and the design of the study together. HG was the project coordinator and mediator in the panels, performed the statistical analyses, interpreting the data and drafted the manuscript. RJ helped with the statistical analyses, interpreting the data and drafting of the manuscript. RW developed the procedures and helped with the interpreting the data and drafting of the manuscript.

## Pre-publication history

The pre-publication history for this paper can be accessed here:



## Supplementary Material

Additional file 1 The CONSORT Checklist presenting all items to be included when reporting the present randomised trial. Click here for file
